# Spent coffee grounds as a suitable alternative to standard soil in ecotoxicological tests

**DOI:** 10.1007/s11356-024-32297-y

**Published:** 2024-02-07

**Authors:** Luís André Mendes, Jorge Domínguez

**Affiliations:** GEA (Grupo de Ecoloxía Animal), Universidade de Vigo, 36310 Vigo, Spain

**Keywords:** Standard guidelines, Organic matter, Earthworms, Circular economy, Ecotoxicology

## Abstract

*Eisenia andrei* is considered in OECD and ISO guidelines to be a suitable replacement for *Eisenia fetida* in ecotoxicological assays. This suggests that other alternative materials and methods could also be used in standard procedures for toxicity testing. The guidelines also favor using less time-consuming procedures and reducing costs and other limitations to ecotoxicological assessments. In recent years, spent coffee grounds (SCG) have been used to produce vermicompost and biochar and as an additive to organic fertilizers. In addition, the physicochemical characteristics of SCG indicate that the material is a suitable substrate for earthworms, with the organisms performing as well as in natural soil. In the present study, a battery of ecotoxicological tests was established with unwashed and washed SCG and a natural reference soil (LUFA 2.2). The test substrates were spiked with different concentrations of silver nitrate. Survival and reproduction of the earthworm *E. andrei* were assessed under different conditions, along with substrate basal respiration (SBR) as a proxy for microbial activity. Seedling emergence and the germination index of *Lepidium sativum* were also determined, following standard guidelines. Exposure to silver nitrate had similar effects on earthworm survival and reproduction, as the estimated effective concentrations (EC_10_ and EC_50_) in unwashed SCG and LUFA 2.2 overlapped. A hormetic effect was observed for SBR in LUFA 2.2 spiked with 12.8 mg/kg but not in unwashed SCG. Both SBR and root development were inhibited by similar concentrations of silver nitrate in washed SCG. The findings indicate that unwashed SCG could potentially be used as a substrate in *E. andrei* toxicity tests and support the eventual inclusion of this material in the standard guidelines.

**Supplementary Information:**

The online version contains supplementary material available at 10.1007/s11356-024-32297-y.

## Introduction

Soil ecotoxicological studies have been reported since the 1960s, and a short-term earthworm survival toxicity test was standardized by the Organization for Economic Co-operation and Development (OECD) in 1984 (OECD 1984). Since then, many standardized test guidelines have been adopted for soil testing, by both the OECD and the International Standards Organization (ISO) (ISO 2002, 2008, 2016; OECD 2009, 2016). These developments have included tests with other species groups and also chronic toxicity testing and evaluation of other parameters such as reproduction and growth (OECD 2016). The guidelines have been frequently updated in the past two decades (OECD 2004, 2016) as knowledge about soil systems has increased and contaminants of emerging concern (CECs) have increasingly been detected.

Some such updates apply to chronic toxicity tests with earthworms, and the standard guidelines have been altered to include *Eisenia andrei*, in addition to *Eisenia fetida* (ISO 2008; OECD 2016), the only species included in the original 1984 earthworm survival test (OECD 1984). These two species are phylogenetically distinct down to their DNA (Pérez-Losada et al. 2005), with different detoxification mechanisms (Jaskulak et al. 2021) and life traits, such as a higher growth and reproduction rate in E*. andrei* when compared to *E. fetida* (Domínguez et al. 2005). Based on the different traits between species, these updates have provided robustness to the information acquired using chronic toxicity tests, focusing on more than one parameter (survival and reproduction), and allowing the assessment of responses that otherwise could not be observed in short exposures.

In addition, the updated guidelines allow the use of natural soil as an alternative to the artificial soil proposed by the OECD, which requires specific reagents and is time-consuming to prepare. This is an important change, as it opens the way to the application of standard guidelines as a tool for assessing soil quality in specific scenarios of local contamination (Romero-Freire et al. 2015a; González-Alcaraz et al. 2018; Römbke et al. 2018). As a result, recent ecotoxicological studies have used LUFA 2.2 soil, a commercially available, standard natural soil (Garcia-Velasco et al. 2016; Lima et al. 2020; Römbke and Martin-Laurent 2020).

However, the need for correct characterization of the natural soil used and the cost associated with the use of commercial alternatives may restrict the application of OECD guidelines in developing countries (Niemeyer et al. 2018).

Spent coffee grounds (SCG) have been shown to be a suitable substrate for earthworms, due to their high organic matter (OM) content. SCG are currently used in vermicomposting systems (Sanchez-Hernandez and Domínguez 2017) and as a raw material for producing biochar (Bomfim et al. 2022; Souza et al. 2022). As such, their application to soil may become more frequent in view of the implementation of directives involving the reuse of waste material in Europe and of their general availability in many countries, due to the widespread production and consumption of coffee around the world (European Commission 2018; United States Department of Agriculture 2022).

The use of SCG in ecotoxicological tests would reflect realistic scenarios and close the gap between laboratory-controlled conditions (established in the OECD standard guidelines) (Fründ et al. 2010) and field conditions (van Gestel 2012). Furthermore, SCG represent a cheaper, ready-to-use alternative, which may be more readily available than the commercial standard LUFA 2.2 soil.

Thus, to assess the viability of using SCG in ecotoxicological tests, LUFA 2.2 soil and unwashed and washed SCG were spiked with silver nitrate (AgNO_3_) as a test contaminant. AgNO_3_ has been widely used in toxicity tests with various soil-dwelling species, including plants, collembolans, enchytraeids, and earthworms (Mendes et al. 2015; Bicho et al. 2016; Tourinho et al. 2021).

Under these conditions, the chronic response of *E. andrei* and substrate basal respiration (SBR), used as a proxy for microbial activity, were determined, together with the seedling emergence of *Lepidium sativum*, following standard guidelines (ISO 2002, 2016; OECD 2016). The data acquired were correlated with the physical–chemical characteristics of the substrates. The study findings can potentially be used for optimizing and updating current OECD standard guidelines, contributing to a more sustainable science and society (Santagata et al. 2021).

## Material and methods

### Substrate origin and physical–chemical characterization

Two different substrates were used in this study: a standard natural soil (LUFA 2.2), acquired from LUFA Speyer (Germany) (widely used in ecotoxicological testing), and spent coffee grounds (SCG), acquired from the university cafeteria, in a homogenous mixture containing different types of coffee (including decaffeinated) from the same manufacturer and kept at room temperature for 1 week prior to use. In addition to this, a third condition was setup, by washing SCG with distilled water in a 1:10 (weight/volume) ratio, in order to mimic a weathering processing and to remove excess caffeine and any other organic compounds that may be harmful to soil organisms.

For each substrate condition, the pH and electrical conductivity were measured in 1:10 (w/V) water extracts. Humidity was measured in samples of soil and SCG dried overnight at 105 °C, and the organic matter content was determined after calcination of samples at 550 °C. Further characterization of soil was provided by the supplier (LUFA 2.2), while SCG has been previously characterized by Sanchez-Hernandez and Domínguez (Sanchez-Hernandez and Domínguez 2017).

### Substrate spiking and extract preparation

Silver nitrate (AgNO_3_) (CAS number 7761–88-8), acquired from Panreac Química (Barcelona), was added to each substrate in solution, at concentrations of 0, 12.8, 32, 80, 200, and 500 mg/kg dry substrate. These concentrations were selected on the basis of previous studies with *E. andrei* (Tourinho et al. 2021). Distilled water was also added to ensure adequate moisture, to a minimum of 50% of the substrate water holding capacity. Substrates were distributed into replicate samples and held at room temperature for 3 days to reach chemical equilibrium.

For preparation of extracts, for each condition, at least 20 g of each substrate was mixed thoroughly in distilled water in a 1:5 ratio (w/V) at 200 rpm for 15 min. The mixture was held at room temperature overnight to ensure phase separation. The supernatant was then vacuum filtered (through filter paper of pore size 8 µm) for use in seedling emergence tests. The exposure conditions and tests performed are summarized in Table 1.

**Table 1:** Substrates, AgNO_3_ concentrations, and test parameters, i.e., toxicity to earthworms, substrate basal respiration, and seed germination, used in the experiments

Substrate	mg AgNO_3_/kg d.w	*E. andrei* chronic toxicity	Substrate basal respiration	*L. sativum* seed germination*
LUFA 2.2 soil	0–12.8–32–80–200–500	✔	✔	✔
Unwashed SCG	0–12.8–32–80–200–500	✔	✔	✔
Washed SCG	0–12.8–32–80–200–500	✔	✔	✔

*Exposed in soil–water extracts

### Chronic toxicity test with Eisenia andrei

The response of *E. andrei* to substrate spiked with AgNO_3_ was assessed following the OECD standard guidelines (OECD 2016), with some modifications. Briefly, groups of 10 mature specimens of *E. andrei*, each with a well-developed clitellum and weighing 335 ± 9 mg (average ± standard error), were thoroughly washed and placed in each replicate containing at least 350 g of substrate. Pre-moistened spent coffee grounds (7 g) were spread across the substrate surface for the first 4 weeks of the test as a food source, and water was replenished weekly. The tests were carried out at 20 ± 2 °C under a photoperiod of 16:8 h light to dark for 8 weeks. After 4 weeks, the surviving adults were removed, counted, washed, and weighed to determine any change in body mass. After 8 weeks, the numbers of juveniles and cocoons were counted with the help of a magnifying lens.

The validity criteria of the test were fulfilled as in the controls (in this case the unspiked LUFA 2.2 soil), the adult mortality was less than 10% after 4 weeks, while the coefficient variation of reproduction was less than 30% and more than 30 juveniles were produced per replicate after 8 weeks.

### Substrate basal respiration (SBR)

In parallel to the toxicity tests with *E. andrei*, an additional test was conducted to assess changes in substrate basal respiration (SBR) (as a proxy for microbial activity), measured as production of CO_2_ per OM weight. After 8 weeks in similar conditions, but without earthworms, the replicate substrates were retrieved and stored for assessment of CO_2_ production (ISO 2002).

The CO_2_ production was measured according to Anderson et al. (1983). Briefly, the replicate substrates were placed in glass jars with small vials containing 20 mL of 20 mM NaOH. The jars were sealed and incubated in darkness for 6 h. Aliquots (5 mL) of the NaOH were then removed and titrated against 10 mM HCl and excess 6N BaCl_2_, with phenolphthalein 1% as a pH indicator, to determine the CO_2_ content. The CO_2_ production rate was calculated on the basis of the amount of organic matter in each sample.

### Lepidium sativum seedling emergence test

*L. sativum* seedling emergence was assessed in extracts obtained from the AgNO_3_-spiked substrates (LUFA 2.2 soil, unwashed SCG, and washed SCG) by adapting the ISO guidelines (ISO 2016). The decision to use extracts was to assess the toxicity of the more readily available Ag that can pass through osmosis to seed tissues. Thus, groups of 30 *L. sativum* seeds were placed in replicate glass Petri dish (ø = 80 mm) lined with Whatman #1 filter paper and filled with 4 mL of substrate extract (1:5 w/V). The dishes were then held in darkness at room temperature for 7 days. The germinated seeds were counted on days 1, 2, 3, and 7, while the root and shoot lengths of each germinated seed were measured after 7 days. A seed was considered germinated when the shoot was longer than 1 mm.

The germination index (GI) was calculated after 7 days on the basis of the relative seed germination (RSG), i.e., the ratio of germinated seeds under test and control conditions, and the relative root growth (RRG), i.e., the ratio of the root length under test and control conditions) (Eq. 1). The relative shoot growth (RShG), i.e., the ratio of the shoot length under test and control conditions) and the root-shoot ratio (RSR) were also calculated.

1 $$GI=RR\;\times\;RSG$$

Equation 1: The germination index (GI) is calculated from the relative root growth (RRG) and the relative seed germination (RSG).

### Estimation of the effect concentration (ECx)

For each parameter measured in each of the ecotoxicological assays, the AgNO_3_ concentration that would cause 10, 20, 50, and 80% effect was estimated using the Toxicity Relationship Analysis Program (TRAP), version 1.30. Except when noted, a 2-parameter logistic model was used as the best-fit approach, with the exposure concentrations transformed by log10.

### Statistical analysis

The Shapiro–Wilk test was used to check the normality of the sample data. To detect significant differences (*p* < 0.05) between the uncontaminated reference LUFA 2.2 soil and each of the uncontaminated tested substrates (washed or unwashed SCG), a Student *T*-test was performed. Two-way ANOVA was used to detect significant differences between concentrations and substrates for each parameter, and a post hoc Dunnett’s test was used to determine differences relative to the control, reference soil (LUFA 2.2).

Multivariate analysis was performed by applying principal component analysis (PCA), including the abiotic factors (pH, OM, and humidity) and parameters measured in the ecotoxicological tests, to identify which factors were most closely correlated and caused significant responses in each substrate. All analyses were performed using SigmaPlot version 14.0.

## Results

### Substrate physical–chemical characterization

The physical–chemical analysis of the three substrates showed significant differences between the soil and SCG in relation to structure, electrical conductivity (EC), and percentage of OM, with higher values of these parameters in the SCG (Table 2). In addition, the washing step was found to interfere with the SCG composition, causing a significant decrease in the values of all chemical parameters, including pH, and an increase in the silt particle content.

**Table 2:** Main characteristics of test substrates prior to spiking and start of the test. Distinct letters over the numbers indicate significant differences between substrates

Substrate	pH	EC (µS/cm)	Humidity (% weight)	OM (% d.w.)	Substrate particle size (mm) (%)			Classification
					2 < *x* < 0.05 (sand)	0.05 < *x* < 0.002 (silt)	*x* < 0.002 (clay)	
LUFA 2.2	6.65 ± 0.07^a^	40 ± 9.5^a^	23 ± 0.3^a^	6.97 ± 1.86^a^	73.9 ± 0.63^a^	15.8 ± 0.54^a^	10.3 ± 1.17^a^	Sandy loam
Unwashed SCG	6.76 ± 0.08^a^	124 ± 11^b^	71.1 ± 2.0^c^	93.4 ± 3.82^b^	90.8 ± 0.49^b^	6.6 ± 0.19^c^	2.6 ± 0.40^b^	Sand
Washed SCG	6.12 ± 0.08^b^	46.9 ± 3.6^a^	64.3 ± 5.3^b^	82.7 ± 12.9^b^	89.0 ± 0.46^c^	8.5 ± 0.05^b^	2.5 ± 0.49^b^	Sand

*SCG* spent coffee grounds, *EC* electrical conductivity, *OM* organic matter, *d.w* dry weight

### Chronic toxicity test with Eisenia andrei

**Fig. 1:**
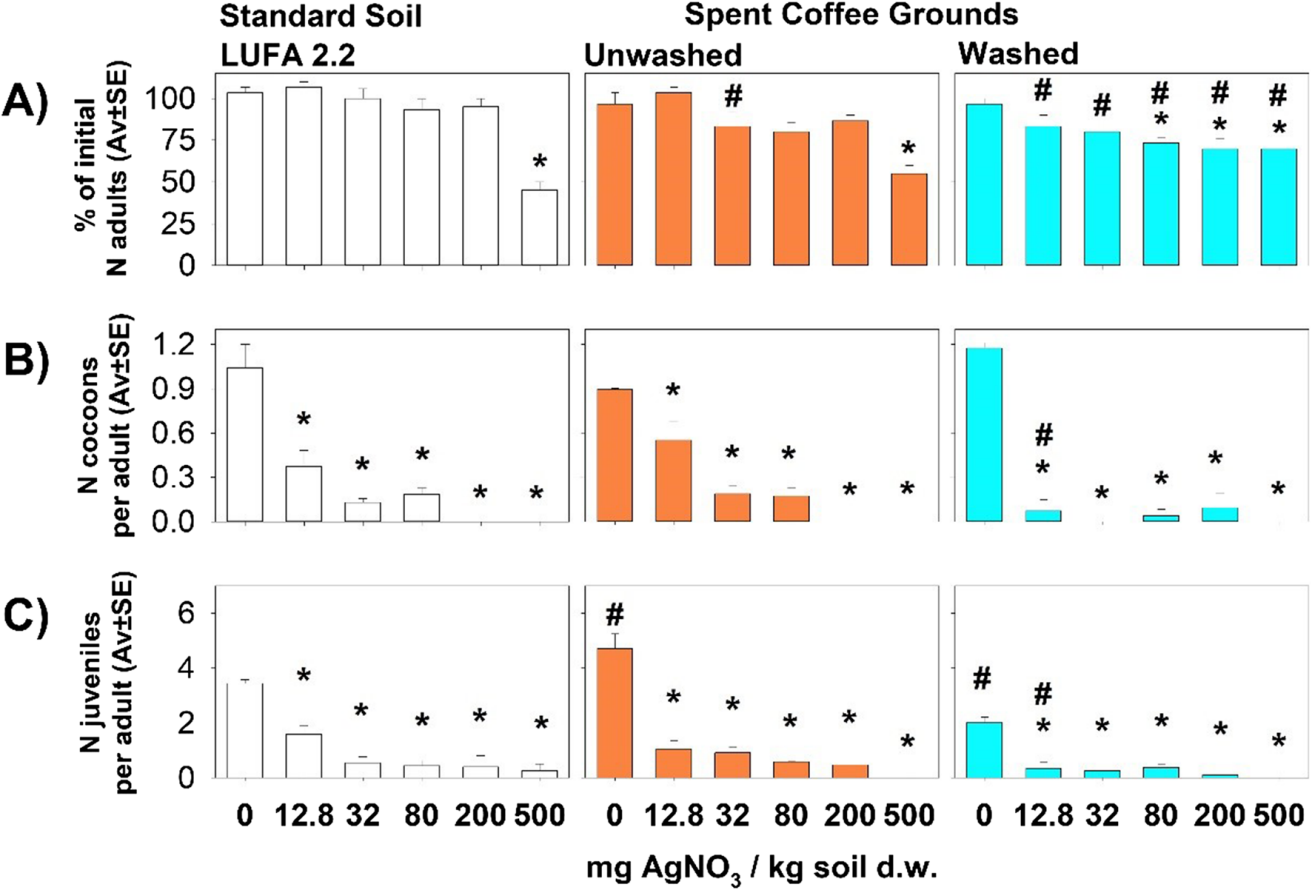
Results of the chronic survival and reproduction tests with E. andrei in LUFA 2.2 soil, unwashed SCG, and washed SCG spiked with AgNO3: **A** number of adult earthworms surviving after 28 days exposure as a percentage of the initial number, **B** number of cocoons produced per adult after 56 days, and **C** number of juveniles produced per adult after 56 days. *Significant difference relative to the control, #significant difference between substrates

Similar results were observed in all tests comparing the different substrates at similar concentrations (Fig. 1). The survival of adult specimens of *E. andrei* was affected at the highest concentrations in all substrates spiked with 500 mg AgNO_3_/kg dry substrate and also in washed SCG spiked with 80 and 200 mg/kg (Fig. 1A).

All AgNO_3_ treatments yielded significant differences relative to the respective controls regarding both the number of juveniles per adult and the number of cocoons per adult (Fig. 1B, C).

In the comparison of different substrates, the number of surviving adults was consistently lower in the AgNO_3_-spiked washed SCG than in the spiked LUFA 2.2 soil. Survival of adult earthworms was significantly lower in the unwashed SCG than in LUFA 2.2 soil only at 32 mg AgNO_3_/kg. Comparison of earthworm reproduction in the unspiked substrates showed a greater number of juveniles per adult in the unwashed SCG than in the LUFA 2.2 soil, but significantly lower numbers in the washed SCG. In addition, at the lowest concentration of AgNO_3_ (12.8 mg/kg), the number of juveniles and cocoons per adult was also significant lower in the washed SCG than in the other substrates.

### Substrate basal respiration (SBR)

**Fig. 2:**
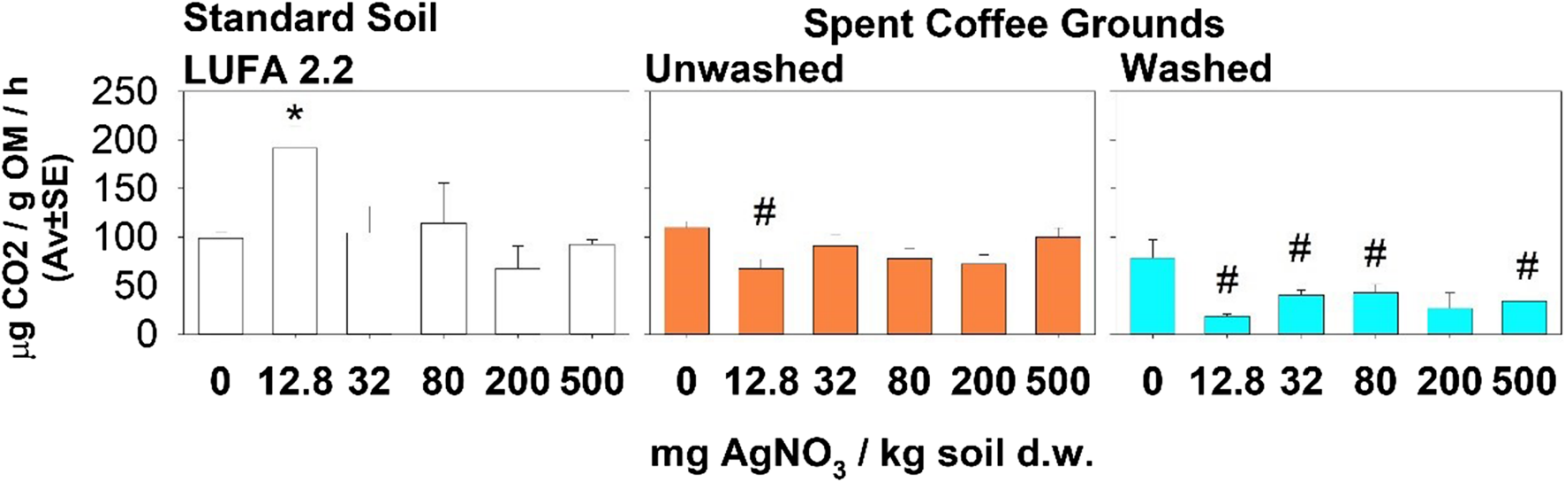
Substrate basal respiration in the LUFA 2.2 soil, unwashed SCG, and washed SCG spiked with AgNO3 after 56 days. *Significant difference relative to the respective control, #significant difference between substrates

Exposure to AgNO_3_ did not induce a concentration-dependent response in microbial activity, measured as SBR, although a significant increase in LUFA 2.2 soil spiked with 12.8 mg AgNO_3_ /kg was observed. Comparison of the different substrates revealed significantly lower CO_2_ production in washed SCG spiked with AgNO_3_ (12.8, 32, 80, and 500 mg/kg) than in the spiked LUFA 2.2 soil (Fig. 2).

### Lepidium sativum seedling emergence test

A slight hormetic effect was observed in germination, namely, in the relative root growth (RRG) and consequently in the germination index (GI), at relative low concentrations (32 mg/kg) in unwashed SCG (Figure S1). On the other hand, a decrease in germination was observed in washed SCG spiked with 12.8 g AgNO_3_ /kg.

### Data and multivariate analysis

#### ***EC***_***50***_*** comparison***

**Fig. 3:**
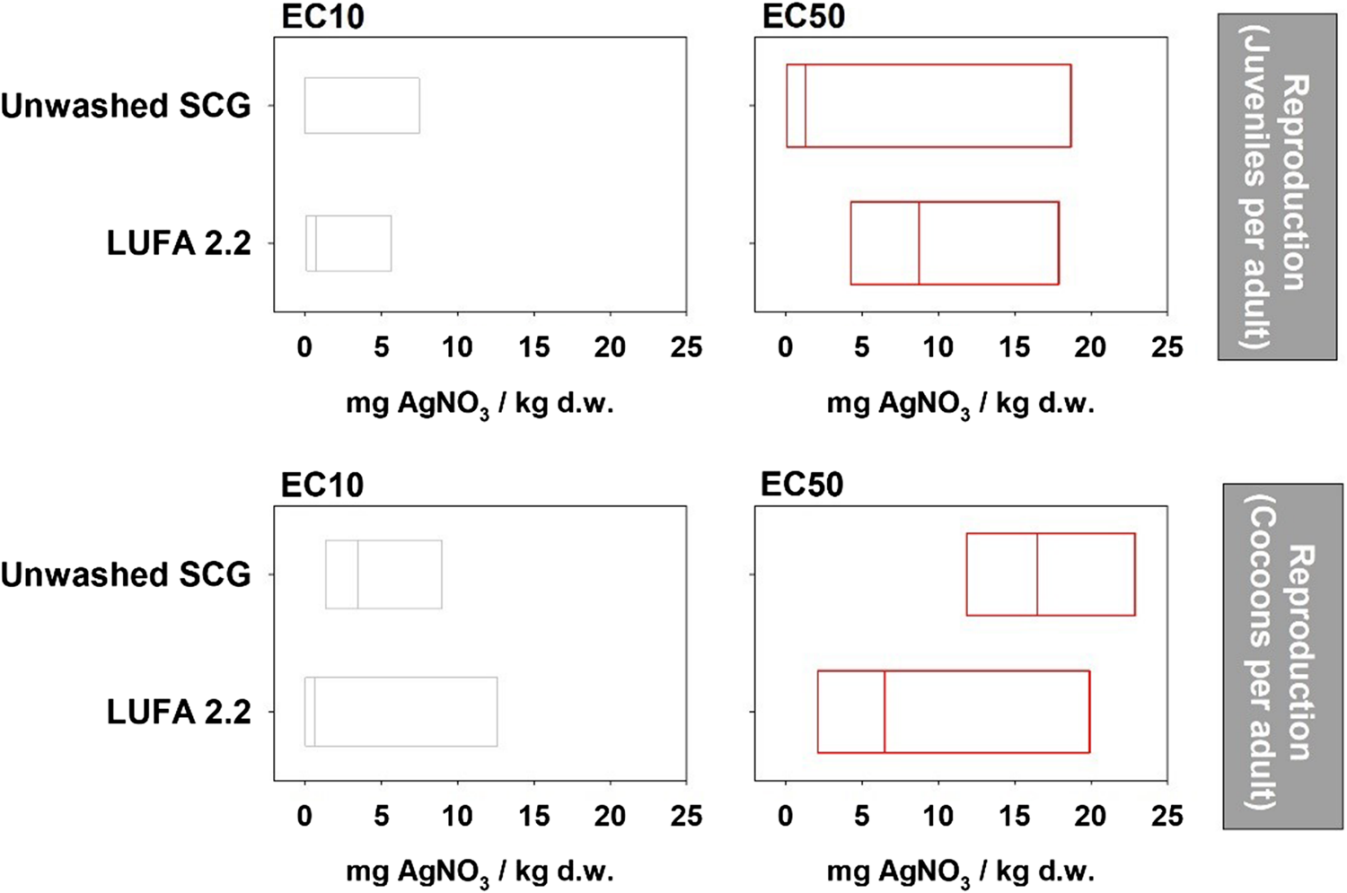
Representation of the estimated 10 and 50% effect AgNO3 concentrations for Eisenia andrei reproduction in unwashed SCG and LUFA 2.2 soil

Comparison of the estimated 10% and 50% effect concentrations for *E. andrei* reproduction revealed a significant overlap of the median and 95% confidence values in LUFA 2.2 and unwashed SCG, indicating a similar level of response in both substrates (Fig. 3).

#### Multivariate analysis

**Fig. 4:**
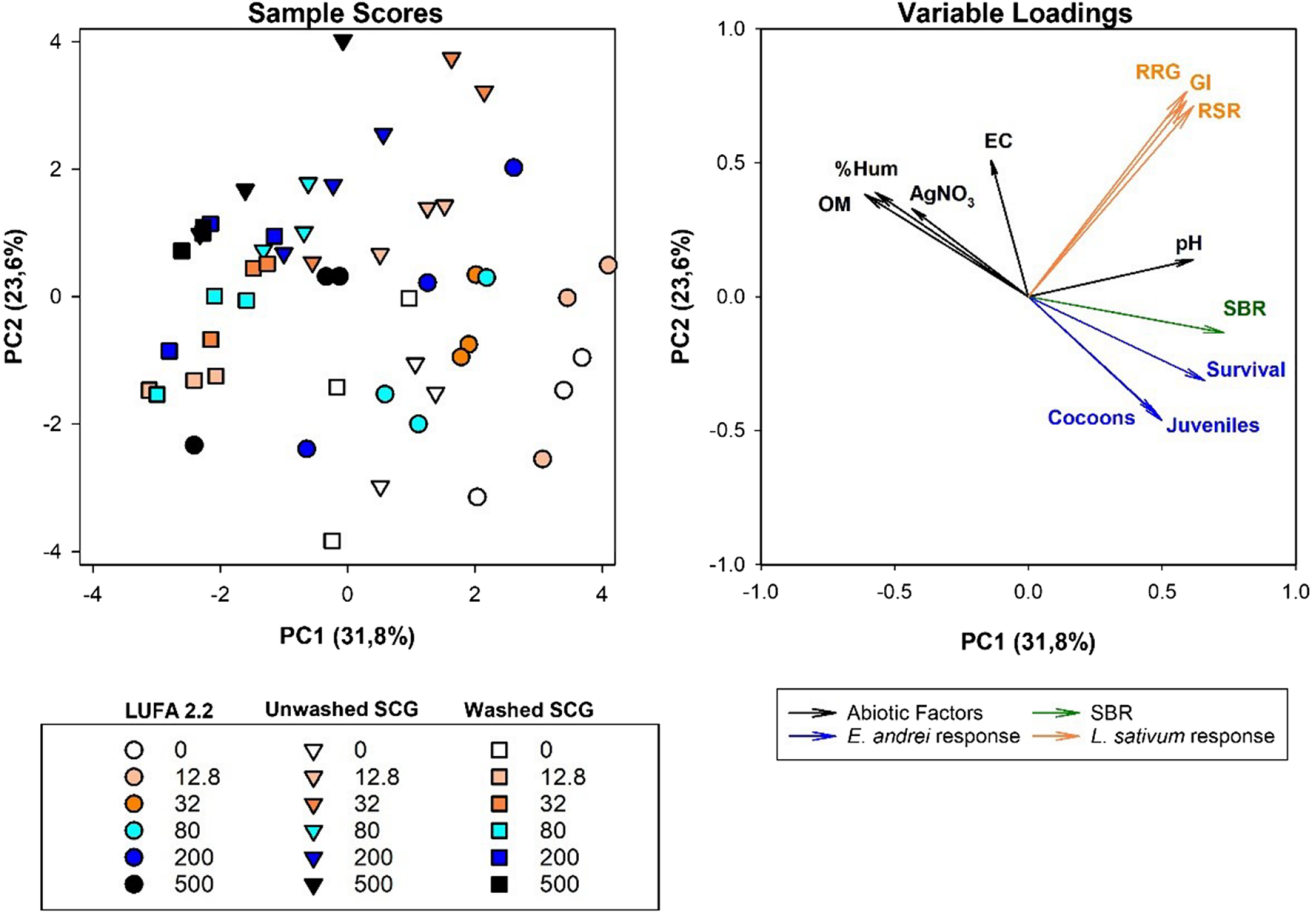
Representation of the principal component analysis, including abiotic factors, the E. andrei response (survival and reproduction), substrate basal respiration (SBR), and the L. sativum response (relative root growth, germination index, and root-shoot ratio) as response variables for the three substrates spiked with different concentrations of AgNO3

The PCA identified 4 in-model principal components (PCs) with eigenvalues greater than 1. Of these, the two main components (PC1 and PC2) accounted for 55.4% of the variance (Fig. 4 and Table S2). The variable loadings indicate that SBR, *E. andrei* survival, *L. sativum* RSR, pH, and %OM were strongly correlated (> 0.6) with PC1 (accounting for 31.8% of the variance), while *L. sativum* parameters (RSG, RSR, and GI) were strongly correlated with PC2 (accounting for 23.6% of the variance). For the sample scores, a cluster of samples of washed SCG spiked with AgNO_3_ was observed on the left side of the figure, showing a clear difference in overall response in the substrate relative to LUFA 2.2 or even unwashed SCG. The variable loading plot also confirmed the previous observations: the *E. andrei* reproduction response vectors (numbers of cocoons and juveniles) were inversely correlated with AgNsO_3_ concentration vector, showing this to be a highly responsive parameter. In addition, *L. sativum* response was not correlated with the *E. andrei* response under these conditions (Table S2).

## Discussion

### Unwashed SCG as an alternative to LUFA 2.2 soil

The study findings did not show any significant differences in *E. andrei* response to AgNO_3_ when exposed in natural LUFA 2.2 soil and in unwashed SCG. In fact, an increase in the earthworm reproductive rate was observed in the unwashed SCG control, suggesting that the initial conditions for *E. andrei* reproduction were more suitable than in the standard soil. This improvement can be explained by the much higher OM content (7% vs. 93%) and humidity (23% vs. 71%) in the unwashed SCG, as *E. andrei* is known to thrive in OM enriched environments and with high levels of moisture (Jänsch et al. 2005), as, e.g., grape marc (Domínguez et al. 2014).

The improved control conditions did not mask any AgNO_3_-induced effects, as the survival and reproduction responses were similar in both substrates, with a significant effect on survival at 500 mg/kg and a clear effect on reproduction in all treatments. This was further supported by the overlapping estimated values of EC_10_ and EC_50_ for reproduction. The EC_50_ value for the number of juveniles in the LUFA 2.2 soil was 9 mg/kg (95% confidence interval of 4–18 mg/kg), which is lower than the value reported in another study in which *E. andrei* was exposed to AgNO_3_ (38 [27–50] mg/kg) (Tourinho et al. 2021).

Similarities between unwashed SCG and LUFA 2.2 were also observed in the SBR. This was unexpected, as previous studies on the effects of SCG amendment to soils showed an increase in SBR due to an increase in OM, as well as containing higher levels of other nutrients such as phosphorus (Cervera-Mata et al. 2018, 2022). On the other hand, the absence of any change in SBR in response to AgNO_3_ in the LUFA 2.2 soil and SCG was somewhat expected, as SBR has previously been shown to be less affected by spiking with metal, i.e., Pb, in highly organic soils (Romero-Freire et al. 2015a).

As for the *L. sativum* response, the use of SCG induced a hormetic effect up to 32 mg/kg for the RRG and RSR parameters, indicating that AgNO_3_ did not have toxic effects in SCG. Another study focusing on the germination response in aqueous solutions containing metals also showed limited toxicity to plants (Romero-Freire et al. 2015a). Both SCG and SCG-derived vermicompost have shown potential for enzymatic remediation, which could explain the low toxicity observed (Sanchez-Hernandez and Domínguez 2017). Other studies testing SCG showed improved growth of radish and tomato seedlings, with success in plant development and in repelling slugs and snails (Horgan et al. 2023). SCG may thus improve the conditions for seedling emergence, although no significant differences between the response in SCG and LUFA 2.2 soil were observed. To our knowledge, this is the first study to include a seedling emergence test for AgNO_3_ toxicity using LUFA 2.2 soil.

### Washed SCG is not suitable as an alternative substrate

While unwashed SCG proved to be an adequate alternative to LUFA 2.2 soil as a test substrate, this was not true for washed SCG. There were significant differences in *E. andrei* response in all parameters tested (survival of adults, number of juveniles, and cocoons). A similar pattern was observed for SBR, as a proxy for microbial activity, which was also significantly lower in washed SCG than in the other substrates. This indicates that the additional washing step did not improve conditions and actually worsened them. The significant decrease in pH (the lowest value of the three) may explain the observed differences, as pH was positively correlated with the substrate basal respiration (Table S2). Prior studies with *E. andrei* exposed to metals (Pb and As) have shown that lower pH can produce greater mortality, probably due to an increase in metal availability in the substrate, and have also shown a reduction in basal respiration in substrates with lower pH (Romero-Freire et al. 2015a, b).

Considering the other physical–chemical characteristics, washed SCG displayed differences in terms of silt and sand content relative to the unwashed SCG. This is important as soil structure (i.e., clay, silt, and sand content) has been shown to play a role in metal toxicity in *E. andrei*, as the metal ions may be more easily adsorbed on larger particles (Van Gestel et al. 2011).

In addition, previous studies have shown that the application of SCG to soil alters the soil structure and porosity (Cervera-Mata et al. 2023), which in turn will affect aeration, microbial colonization, growth, and activity (Quilliam et al. 2013). The reduced SBR (microbial activity) observed in washed SCG may indicate that the aggregation ability may have been affected by the washing step.

The unsuitability of washed SCG was further demonstrated by the *L. sativum* germination test, as the decrease in GI at 12.8 mg/kg reversed the hormesis observed for unwashed SCG and LUFA 2.2 soil, indicating a loss of beneficial properties. This finding is consistent with those of a recent study using dried/aged SCG and torrefacted SCG, in which aqueous extracts of dried SCG inhibited GI (Jeníček et al. 2022).

### Future perspectives

The study reported here compared the response of three representative components of the soil system (invertebrates, microbes, and plants) in three substrates spiked with AgNO_3_ in order to assess whether SCG, a readily available waste product, could be used in ecotoxicological assays, according to standard guidelines, as an alternative to the widely used natural LUFA 2.2 soil.

The data obtained in the *E. andrei* chronic toxicity test appeared promising in relation to the use of unwashed SCG, as the reproductive output increased under control conditions. This would allow better distinction between different levels of response that may occur, facilitating estimation of effective concentrations and more accurate assessment of sub-lethal effects to soil organisms. These findings should be therefore considered in the experimental design for risk assessment following standard guidelines (Römbke et al. 2018).

On the other hand, SBR was expected to be much higher in unwashed SCG than in LUFA 2.2 soil, based on the higher OM content. In addition, the lack of a response in the *L. sativum* germination test also limits the potential use of SCG as a substrate for such tests.

This suggests that the quality rather than amount of OM is a key factor, as the type of OM (e.g., humic acids) can interact with other components of the matrix and modulate the toxicity (Barbero et al. 2021). Further evidence that factors other than OM content was obtained in this manuscript, as washed SCG was shown to be unsuitable as an alternative substrate, owing to lower pH and other properties. This was demonstrated by the lack of correlation between the parameters tested in *L. sativum* and the abiotic factors measured (Table S2). This remains a point of discussion in regard to ecotoxicological assays (van Gestel 2012; Romero-Freire et al. 2015a). Other studies considering changes in soil properties and plant development have observed that use of SCG increased the nutrient and metal contents, along with C:N dynamics (Cervera-Mata et al. 2018, 2022). This should be further explored to understand the microbial and seedling response to spiked SCG.

In the present study, ecotoxicological tests were performed using inorganic compounds. To further support the use of unwashed SCG as an alternative substrate, studies must be performed with other CECs, namely, those of organic nature, e.g., carbendazim, imidacloprid, and many other compounds that are still widespread in soil ecosystems and have widely been tested (van Gestel et al. 2017; Daam et al. 2020).

## Conclusion

This study focused on testing the suitability of SCG as an alternative to commercially available standard soils in ecotoxicological assays following current standard guidelines, aiming to reduce costs and avoid time-consuming processes that do not reflect real scenarios of contamination. It was found that while unwashed SCG can be used to assess the response of *E. andrei* in chronic toxicity tests, the same is not true for substrate basal respiration or *L. sativum* germination. Further studies should be performed with other types of contaminants with a view to the inclusion of SCG as a test substrate in the current standard guidelines.

### Supplementary Information

Below is the link to the electronic supplementary material.

Supplementary file1 (DOCX 542 KB)

## Data Availability

The datasets generated and analysed during the current study are available from the corresponding author on reasonable request.
