# Supplementary material for: Spent coffee grounds as a suitable alternative to standard soil in ecotoxicological tests
Source: Environ Sci Pollut Res Int. 2024 Feb 7;31(11):16725–34. doi: 10.1007/s11356-024-32297-y (PMC10894160; doi:10.1007/s11356-024-32297-y)
Supplement: Supplementary file 1 — Supplementary file1 (DOCX 542 KB) [file 11356_2024_32297_MOESM1_ESM.docx]

# **Supplementary Information**


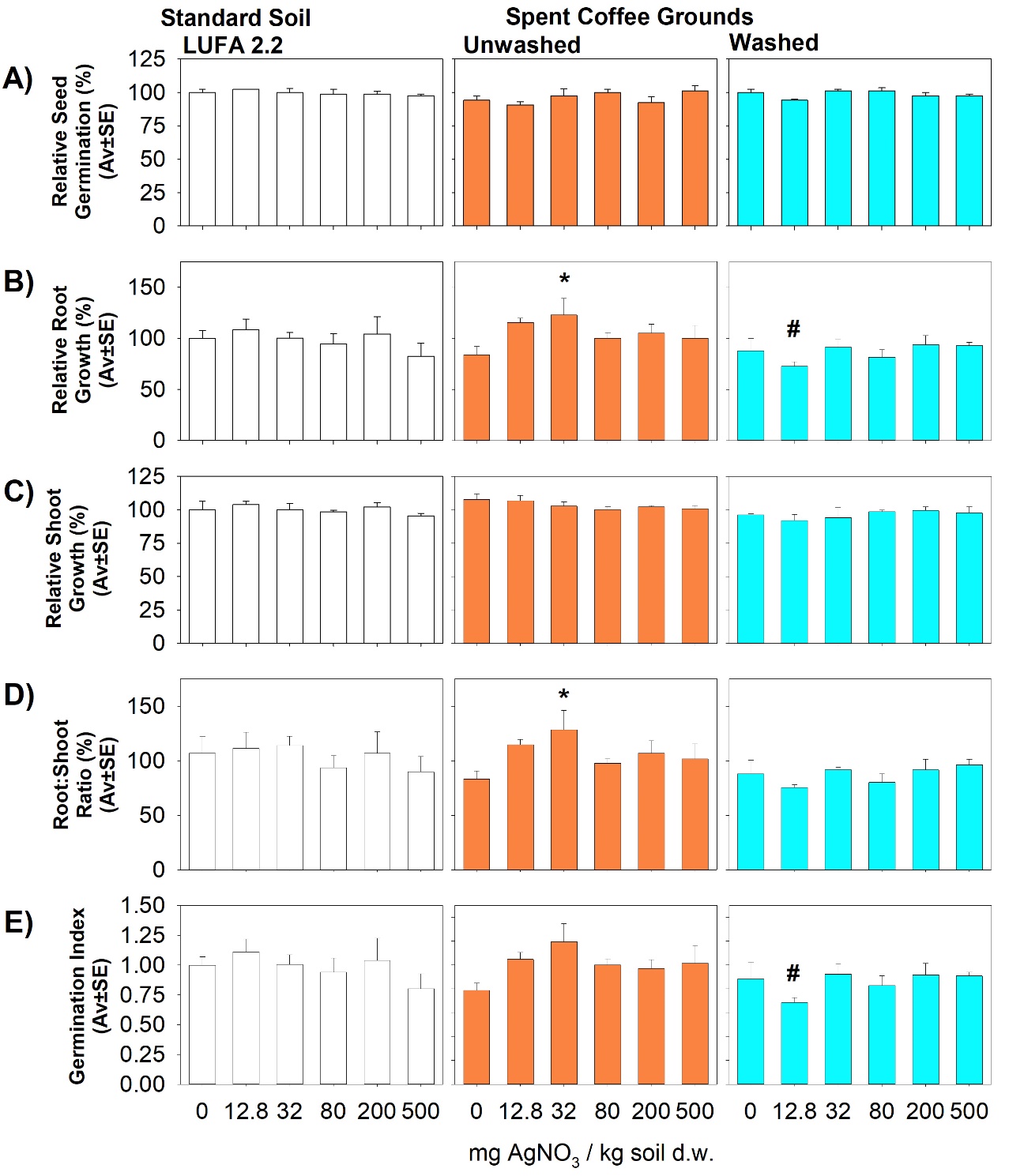
Figure S1: Results of the seedling emergence test with *L. sativum* in LUFA 2.2 soil, unwashed and washed spent coffee grounds spiked with AgNO_3_, after 7 days: A) Relative Seed Germination, B) Relative Root Growth, C) Relative Shoot Growth, D) Root:Shoot Ratio, E) Germination Index. * - Significant difference relative to the respective control, # - significant difference between substrates.


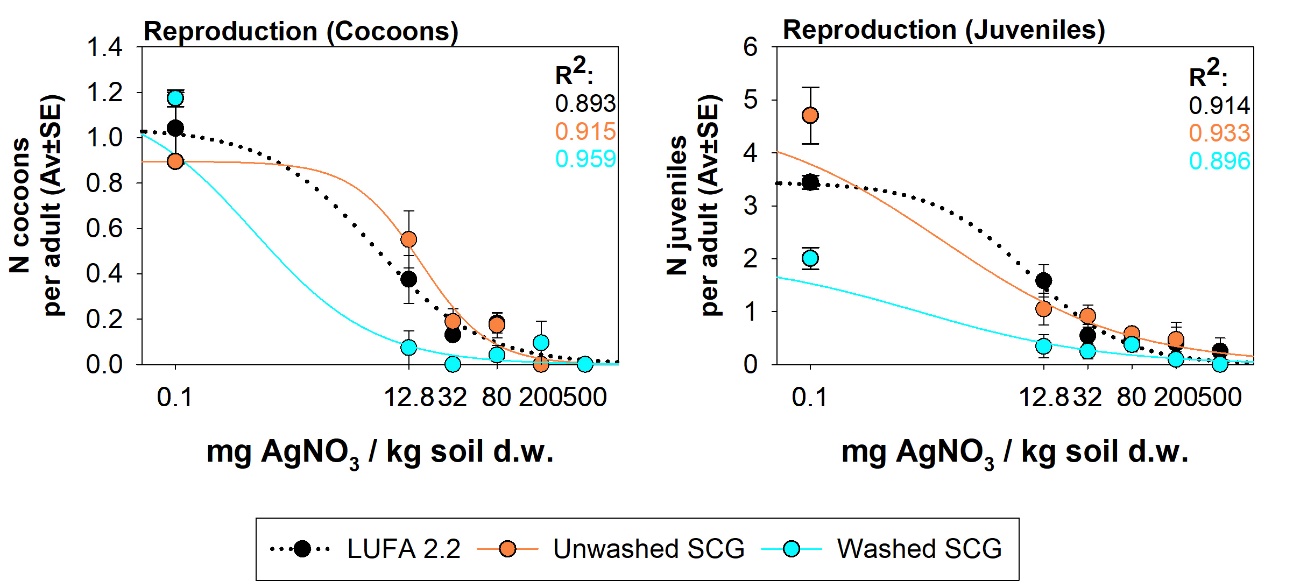


Figure S2: Estimated effect for *E. andrei* reproduction (cocoons and juveniles) in LUFA 2.2 soil, unwashed SCG and washed SCG spiked with AgNO_3_.

Table S1: Further characterization of LUFA 2.2 soil and Spent Coffee Grounds (SCG) substrate batches.

|  | LUFA 2.2 soil | Spent Coffee Grounds |
| --- | --- | --- |
| pH | 6.65 ± 0.07 | 6.76 ± 0.08 |
| EC (µS/cm) | 40 ± 9.5 | 124 ± 11 |
| % Organic Matter | 6.97 ± 1.86 | 93.4 ± 3.82 |
| % Total Carbon | 2.21 ± 0.54 | 53.2 ± 0.76 |
| % Total Nitrogen | 0.20 ± 0.06 | 2.34 ± 0.01 |
| C/N Ratio | 8.6 ± 0.31 | 22.7 ± 0.34 |
| P (mg/kg d.w.) | 292 ± 40 | 1100 ± 80 |
| EC – Electrical Conductivity; C – Carbon; N – Nitrogen; P – Phosphorus; d.w. – dry weight | | |

Table S1: Principal Component Analysis (PCA) in-model components, variable loadings and explained variance. Correlation values over |0.6| are highlighted in bold.

|  |  | PC1 | PC2 | PC3 | PC4 |
| --- | --- | --- | --- | --- | --- |
|  | Variance (%) | 31.8 | 23.6 | 19.5 | 10.9 |
| *E. andrei* response | Survival | **0.659** | -0.313 | 0.377 | -0.323 |
|  | Reproduction (Juveniles) | 0.499 | -0.462 | 0.558 | 0.238 |
|  | Reproduction (Cocoons) | 0.476 | -0.437 | 0.562 | 0.026 |
| Microbial activity | SBR | **0.732** | -0.135 | -0.142 | 0.413 |
| *L. sativum* response | RRG | 0.593 | **0.767** | 0.038 | -0.173 |
|  | RSR | **0.618** | **0.712** | -0.049 | -0.172 |
|  | GI | 0.591 | **0.731** | -0.032 | -0.224 |
| Abiotic factors | AgNO_3_ concentration | -0.434 | 0.328 | -0.544 | 0.451 |
|  | pH | **0.616** | 0.138 | -0.022 | **0.617** |
|  | EC | -0.139 | 0.509 | 0.557 | 0.523 |
|  | % Humidity | -0.571 | 0.389 | **0.699** | 0.013 |
|  | % OM | **-0.611** | 0.381 | **0.672** | -0.026 |

Table S2: PCA Variables Correlation Matrix. Correlation values over |0.6| are highlighted in bold.

|  | Surv. | Reprd. (Juv.) | Reprd. (Coc.) | SBR | RRG | RSR | GI | AgNO_3_ conc. | pH | EC | % Hum. | % OM |
| --- | --- | --- | --- | --- | --- | --- | --- | --- | --- | --- | --- | --- |
| Survival | **0.779** |  |  |  |  |  |  |  |  |  |  |  |
| Reproduction (Juveniles) | **0.607** | **0.830** |  |  |  |  |  |  |  |  |  |  |
| Reproduction (Cocoons) | **0.654** | **0.759** | **0.735** |  |  |  |  |  |  |  |  |  |
| SBR | 0.337 | 0.446 | 0.338 | **0.745** |  |  |  |  |  |  |  |  |
| RRG | 0.221 | -0.078 | -0.035 | 0.254 | **0.971** |  |  |  |  |  |  |  |
| RSR | 0.221 | -0.089 | -0.049 | 0.292 | **0.940** | **0.920** |  |  |  |  |  |  |
| GI | 0.221 | -0.114 | -0.062 | 0.246 | **0.948** | **0.925** | **0.934** |  |  |  |  |  |
| AgNO_3_ concentration | **-0.739** | -0.564 | **-0.644** | -0.098 | -0.105 | -0.085 | -0.100 | **0.795** |  |  |  |  |
| pH | 0.155 | 0.379 | 0.237 | **0.690** | 0.364 | 0.374 | 0.327 | 0.069 | **0.780** |  |  |  |
| EC | -0.210 | 0.131 | 0.038 | -0.033 | 0.239 | 0.160 | 0.156 | 0.16 | 0.296 | **0.862** |  |  |
| % Humidity | -0.239 | -0.072 | -0.049 | -0.564 | -0.016 | -0.112 | -0.078 | 0.001 | -0.305 | **0.673** | **0.967** |  |
| % OM | -0.260 | -0.112 | -0.080 | **-0.605** | -0.040 | -0.134 | -0.098 | 0.013 | -0.354 | **0.639** | **0.967** | **0.971** |
